# A journey through wheat meiosis: from polyploid origins to genome stability and exceptional fertility

**DOI:** 10.1098/rspb.2026.1552

**Published:** 2026-11-11

**Authors:** Azahara C. Martin, Graham Moore

**Affiliations:** ^1^ Crop genetics, Institute for Sustainable Agriculture, Córdoba 14004, Spain; ^2^ Crop genetics, John Innes Centre, Norwich Research Park, Norwich NR4 7UH, UK

**Keywords:** wheat, meiosis, *Ph1*, *ZIP4*, crossover, synapsis

## Abstract

Historians have long noted that human civilization owes much to the exploitation of a remarkable genetic event: the fusion of three wild grasses in the Middle East over 10 000 years ago that gave rise to bread wheat. Today, bread wheat nourishes around 4.5 billion people on the planet. Despite its hybrid origins, wheat behaves cytologically as a diploid during meiosis, producing balanced gametes and maintaining exceptional fertility. A recent step-change in wheat genetic resources has enabled the identification of a single meiotic gene, *ZIP4*, as the key factor responsible for stabilizing this complex polyploid genome. In this Perspective, we trace the journey from classical cytogenetics to modern genomics that culminated in the identification of this gene, summarize recent insights into its crucial role during meiosis, and discuss its relevance for addressing the future challenges posed by climate change and global food‑security.

## 1. Introduction

Bread wheat underpins food security for much of the world, yet its continued success depends on sustaining productivity in the face of increasing pressures from rising temperatures, water scarcity, the need to reduce pesticide and fertilizer inputs, and the demand for more resilient agricultural systems. Demand for wheat is also shaped by demographic and social changes. Although global population growth is slowing with a plateau expected later this century, rapid urbanization, rising per capita consumption, and increasing geopolitical instability continue to drive the need for resilient and adaptable wheat production systems. Meeting these challenges requires both the maintenance of stable yields and access to increased genetic diversity for future improvements. In the UK, annual wheat yield was around 1 tonne in the middle of the 19th century, 2.5 tonnes by the 1940s and an average of 8 tonnes per hectare by 1980 [1]. However, within Northern Europe, yield increases have been slowing since the mid-1990s [1]. Since the 1940s, interest in using wheat wild relatives to supply useful agronomic traits, such as novel disease resistance, improved nitrogen-use efficiency, and tolerance to heat and drought has been growing, although to date, only a small fraction of this available genetic diversity has been exploited [2,3]. Recently, more systematic breeding approaches have been adopted to supplement the established strategy of crossing only elite wheat varieties, in order to exploit the vast array of genetic diversity available in wild relatives, as well as that within old wheat landraces (developed by farmers in previous centuries using traditional practices) [3].

The evolutionary and domestication history of modern wheat is rooted in the Fertile Crescent, a region spanning parts of the eastern Mediterranean and Mesopotamia, where early farming communities began to domesticate wild grasses. Tetraploid (AABB) pasta wheat arose through the hybridization of two ancestral wild grasses, *Triticum urartu* (the A genome progenitor) and an unknown member of the Sitopsis group (the B genome progenitor) around 0.3–0.5 million years ago. Hexaploid (AABBDD) bread wheat arose much more recently, approximately 10 000 years ago, when tetraploid wheat hybridized with a third grass, *Aegilops tauschii* (the D genome progenitor), forming a species with three ancestral genomes. Each of these ancestral genomes retains the basic number of seven chromosome pairs, as occurs in diploid relatives, such as barley, rye and other associated wild grasses. In these diploid species, each of the seven pairs is composed of identical chromosomes (which recognize each other during meiosis), known as *homologues*. By contrast, tetraploid and hexaploid wheat contain a total of 14 and 21 homologous chromosome pairs, respectively. In addition to its true homologue, each chromosome has highly similar counterparts inherited from the other ancestral genomes. These counterparts are traditionally termed *homoeologues*, but for clarity we refer to them in this Perspective as *related chromosomes*. Thus, chromosomes can be organized into seven groups of related chromosomes. For example, Group 3 comprises 3A and 3B chromosome pairs in tetraploid wheat and 3A, 3B and 3D chromosome pairs in hexaploid wheat. Genome sequencing revealed that within each Group, the related chromosomes retain a similar gene order and content [4]. As such, the corresponding genes on these related chromosomes exhibit 95% identity and similar expression patterns, compared with the >99% identity exhibited on their homologues [4,5].

A remarkable feature of bread wheat is that, despite its origin as a hybrid arising from three distinct grass species, it undergoes meiosis as if it were a diploid. Chromosomes are recognized with extraordinary fidelity, each chromosome only finding and exchanging genetic material with its true ancestral partner, rather than with the related chromosomes arising from other progenitor genomes. This diploid-like behaviour is central to the stability and long-term fertility of wheat. Without such precision, the hybrid genome would generate unbalanced gametes, compromising fertility and threatening the viability of a crop that has sustained agriculture for millennia. Wheat genome sequencing confirms the remarkable stability of this polyploid [4] with few rearrangements; by contrast, other polyploids, such as hexaploid oat show extensive translocations and recombination between related chromosomes throughout the genome [6]. Yet, this evolutionary success presents a scientific and practical paradox. The same mechanism that stabilizes the polyploid wheat genome also restricts recombination with its wild relatives. Such relatives harbour extensive genetic diversity for traits, such as disease resistance, nutrient use efficiency and tolerance to heat and drought, required for modern breeding. Since pairing and recombination of related chromosomes is so effectively prevented in wheat, introgression of beneficial alleles from wild relatives has often been slow and technically demanding. Understanding the molecular basis of this cytological stability is therefore not only a fundamental biological question but also a key bottleneck in mobilizing genetic diversity for crop improvement.

The search for this critical mechanism began with classical cytogenetics. Early work established that both tetraploid and hexaploid wheat form regular pairs of homologous chromosomes at meiosis, despite carrying multiple related genomes whose chromosomes could otherwise pair incorrectly [7]. The discovery that a single genetic locus—later termed *Ph1*—was largely responsible for enforcing this specificity shaped decades of research and debate (reviewed in [8–10,11]). However, the size and complexity of the wheat genome, together with the limited availability of molecular tools, meant that progress towards a molecular identity was slow. This landscape changed with the emergence of high-quality reference genomes, diverse mutant resources and comparative cereal genomics. These advances enabled a re-examination of the *Ph1* region, ultimately leading to the identification of a single duplicated copy of the meiotic gene *ZIP4* on chromosome 5B (termed *ZIP4-5B*) within the classical *Ph1* interval, as the key factor responsible for stabilizing this complex polyploid genome [12]. This discovery offered a long-sought molecular explanation for the ability of wheat to behave as a diploid during meiosis.

In this Perspective, we revisit the historical path followed from cytogenetics to genomics, in the quest to understand this unusual behaviour of wheat chromosomes during meiosis. We focus on the key conceptual insights that emerged from the identification of *ZIP4-5B* and subsequent genetic studies, including the generation of ‘separation-of-function’ variants in which the advantageous promotion of correct homologue synapsis is uncoupled from the unhelpful restriction of related chromosome exchange. Throughout, we aim to articulate lessons that reach beyond wheat, namely principles of homologue recognition, pairing and recombination control, which are relevant more broadly to the study of polyploid evolution and chromosome biology.

Finally, we consider the practical implications. If a single duplicated meiotic gene can shape the stability of a complex polyploid, then precise modulation of that gene may help unlock genetic diversity without reducing fertility, providing new opportunities for the targeted introgression of advantageous traits. Modulating this single meiotic gene may also facilitate more efficient introgression via the redistribution of recombination sites along chromosomes. By focusing on these selective, concept-driven advances, rather than attempting to provide a complete account of the field, our goal is to show how wheat, long valued as a crop, also illuminates the fundamental principles of meiosis in complex genomes, providing broader insights into chromosome biology and polyploid evolution, while highlighting why those principles matter for future crop improvement.

## 2. From cytogenetics to genomics: the search for the wheat stabilizing mechanism

Before revisiting classical cytogenetics, we briefly outline the key events of meiosis, as this terminology will be used throughout the article. Meiosis is the specialized cell division in which chromosome number is halved to form gametes for sexual reproduction. Its early stages involve the recognition and pairing of homologous chromosomes, followed by synapsis, the intimate alignment of each pair along its length, mediated by the synaptonemal complex (wheat being one of the first species in which this complex was observed [13]). Genetic exchange or recombination (crossover formation) occurs during prophase I, but its consequences are typically evaluated at metaphase I, when pairs of homologues are visible as bivalents connected by crossover (CO) sites. At least one CO must form between every pair of homologues to ensure accurate segregation; this is known as *the obligate CO*. Chromosomes lacking any COs appear as individual chromosomes known as univalents [14,15].

In polyploid species such as wheat, meiosis is particularly challenging because each chromosome must identify its true homologue among highly similar related chromosomes, to ensure balanced segregation and fertility. Despite this challenge, polyploid wheat meiosis is exceptionally regular: at metaphase I, the 42 chromosomes of hexaploid wheat consistently form 21 true homologous bivalents [16], even though each chromosome also has the potential to synapse and recombine with one or more of its other four related chromosomes [17,18].

The question of how bread wheat maintains orderly meiosis, despite carrying three related ancestral genomes, has intrigued researchers for more than half a century. Early studies revealed that when different wheat wild relatives are crossed with each other, related chromosomes in F1 hybrids (first generation progeny) are able to CO freely during meiosis, despite gene identity being as low as 85% [18]. By contrast, when wild relatives are crossed with wheat itself, there is little CO between wheat and wild relative chromosomes ([19], reviewed in [20]). These contrasting outcomes led to the realization that some form of genetic system must have evolved during wheat polyploidization, suppressing CO between related chromosomes [21].

In the 1950s, to further dissect the genetic basis of this observation, Sears developed a remarkable set of nulli-tetrasomic lines: wheat plants missing both copies of a given chromosome (nullisomic) but carrying four compensating copies of a related chromosome (tetrasomic) [7,22]. These lines enabled chromosome-specific functional analysis. Riley [21] & Sears [23] independently observed that loss of chromosome 5B led to the induction of CO between related chromosomes. Riley further recognized that chromosome 5B must therefore carry a genetic control mechanism stabilizing the wheat polyploid, which he named *Pairing homoeologous 1 (Ph1)* [21,24]. Their foundational experiments showed that removing chromosome 5B disrupts this control, allowing meiotic CO between related chromosomes which would normally be prevented.

Sears [25] subsequently refined the location of *Ph1* by screening an X-ray mutagenized population and identifying a line (termed *ph1b*) carrying a deletion on chromosome 5B that permitted CO between related chromosomes. A corresponding *Ph1* deletion mutant (*ph1c)* was also identified in tetraploid wheat a short time later [26]. The *ph1b* deletion is now known to be 59.3 Mb in size, spanning 1187 genes [27]. Remarkably, for the last 40 years, this mutant has been exploited worldwide in breeding programmes to facilitate the transfer (introgression) of agronomic traits from more than 50 wild and related species into wheat [20], saving the US economy alone more than 1 billion dollars. This is one of the best examples of the exploitation of meiotic studies leading to a major applied outcome.

The availability of chromosome 5B deletion lines and the *ph1b* mutant stimulated decades of investigation into the mode of action of *Ph1*, leading to numerous hypotheses involving chromosome organization, pairing, synapsis and CO control (reviewed in [8–9,28–35]). However, these studies necessarily relied on the absence of the whole chromosome 5B, 5BL chromosome arm or on the large *ph1b* deletion, genetic backgrounds that are now known to induce chromosome rearrangements and genome instability (figure 1). As a consequence, interpretation of the associated meiotic phenotypes was often indirect, making it difficult to distinguish primary effects of *Ph1* from secondary consequences of the underlying chromosomal alterations. Only once the gene underlying *Ph1* was identified could its molecular function be rigorously dissected. However, the molecular identity of the *Ph1* locus remained elusive for decades. The locus spanned a large, complex and gene-dense region, making classical positional cloning extremely challenging in the vast and highly repetitive wheat genome (the bread wheat genome being fivefold larger than the human genome). Consequently, for many years, progress relied primarily on cytological phenotypes and mutagenesis rather than molecular analysis.

**Figure 1.**
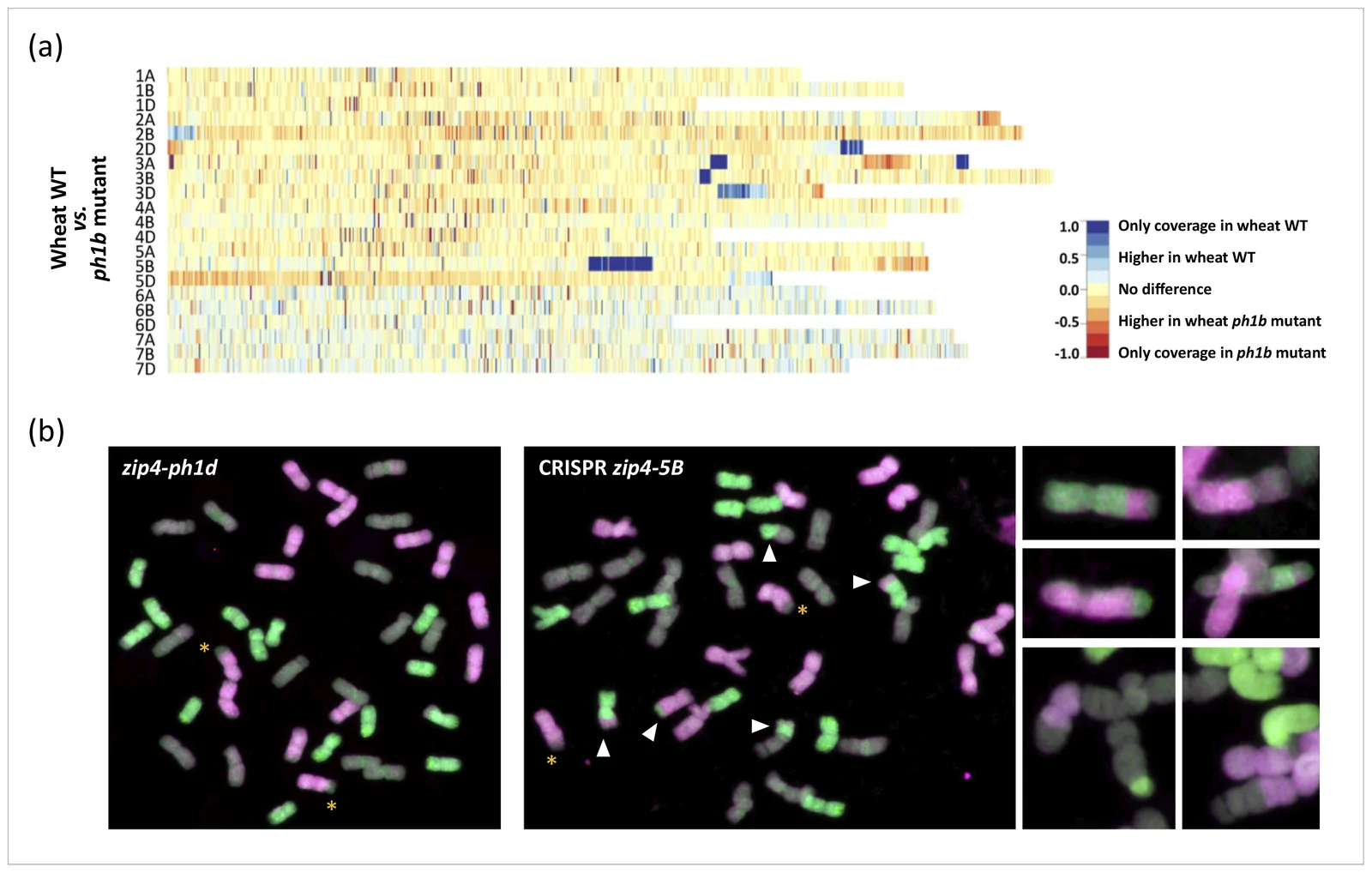
Chromosome instability in the *ph1b* and Clustered Regularly Interspaced Short Palindromic Repeats (CRISPR) *zip4-5B* mutants compared with the *zip4-ph1d* mutant. (a) Genome‑wide comparison of gene expression in wild‑type wheat (*ZIP4‑5B*) and the *ph1b* mutant. Chromosomes (1A–7D) are shown along the vertical axis. The *ph1b* mutant displays multiple deletions (visualized in dark blue) and other chromosomal rearrangements, indicating widespread genome instability. Adapted from [27]. (b) Genomic *in situ* hybridization images illustrating chromosome organization. The A genome is shown in magenta, the B genome in grey and the D genome in green. The ancient translocation T4A·7B is indicated by yellow asterisks. The separation‑of‑function *zip4‑ph1d* mutant (left) shows largely normal chromosome composition, whereas the CRISPR *zip4-5B* mutant (centre) displays extensive chromosome rearrangements and intergenomic exchanges, indicated by arrowheads. Panels on the right show representative examples of rearranged chromosomes in the CRISPR *zip4-5B* mutant.

A key turning point came in the 1990s with the discovery of conserved synteny across cereal genomes. Working with colleagues, we observed that the gene order of rice, a genome approximately 40 times smaller than wheat, mirrored the wheat genome in large chromosomal blocks [36,37]. It soon became evident that this conservation extended across major cereals, enabling the reconstruction of a 60-million-year-old ancestral cereal genome from which modern cereals evolved [37]. This was confirmed in 1995, when we identified equivalent chromosome segments with similar gene order in all agriculturally important cereals, such as sorghum, maize, rye, oats, barley and others, allowing these genomes to be assembled into a single, coherent map [37]. Recognizing the significance of this discovery, we repurposed the term ‘Synteny’ (previously used for a different concept in the human-mammalian field) to describe this new cereal concept, a usage now widely accepted. This insight made it possible to use the compact rice genome as a guide for navigating the wheat genome, accelerating marker development and gene identification across cereals. This *synteny revolution* laid the groundwork for the comparative genomic approaches that would later be essential for resolving *Ph1*.

Further delimitation of the *Ph1* locus required the development of 10 mutagenized populations over a 10 year period, screened for lines carrying chromosome 5B deletions [38]. Cereal Synteny was used to develop molecular markers delineating the boundaries of deleted areas, and lines were crossed with wild relatives to confirm loss of *Ph1* activity [39]. This approach enabled us to narrow the *Ph1* locus to an approximately 0.5 Megabase (Mb) region of chromosome 5B, containing a cluster of cell-cycle-dependent kinases involved in replication, disrupted by an inserted segment of heterochromatin and a meiotically expressed gene of unknown function, both of which had been duplicated from different regions of chromosome 3B [27,39,40]. Unfortunately, at that point in time, attempts to silence the unknown meiotic gene using RNA interference failed to produce viable plants.

Over the following decade, a new generation of genomic and genetic resources emerged that finally made it possible to interrogate the unknown meiotic gene within the *Ph1* interval [41]. These advances included: the sequencing of multiple tetraploid and hexaploid wheat genomes [42,43]; the creation and full sequencing of Targeting Induced Local Lesions in Genomes (TILLING) populations spanning the complete gene complement in both tetraploid and hexaploid ploidy backgrounds [44]; large-scale RNA sequencing (RNA‑seq) datasets covering more than 1 000 tissues, including enriched meiocyte samples [5,27]; the establishment of CRISPR-based targeted mutagenesis in wheat [45]; speed‑breeding systems enabling rapid generation cycling and efficient stacking of mutations [46]; and the development of integrated genomic–phenotypic databases that made these resources readily searchable across the community [47]. Convergence of these advances allowed several groups to return to the refined *Ph1* interval, with far more powerful strategies than had been available during the initial mapping work. For the first time, candidate genes within the region could be systematically tested using complementary genomic, mutant and functional approaches. During this same period, a meiotic gene named *ZIP4* was identified and characterized in budding yeast, rice and *Arabidopsis*, which we realized had high protein sequence homology with the previously uncharacterized meiotic gene embedded in the 0.5 Mb *Ph1* region of wheat chromosome 5B [48–50]. This led to an unexpected insight: the wheat *Ph1* deletion contains a duplicated and diverged copy of *ZIP4*. This variant is formally designated *TaZIP4-B2*, but here we refer to it as *ZIP4‑5B* for clarity. *ZIP4‑5B* rapidly emerged as the leading candidate for the long-sought-after *Ph1* function.

## 3. The master controller *ZIP4-5B*

The identification of wheat *ZIP4‑5B* finally allowed us to move from mapping to mechanism: after six decades of searching, we now had a tangible candidate for the locus stabilizing wheat meiosis. Yet understanding why this duplicated gene had such a profound effect required looking closely at its origin, expression and function.

### (a) A duplicated gene with a new purpose

Hexaploid wheat carries four copies of *ZIP4*: three on the Group 3 chromosomes (3A, 3B and 3D) and a fourth on chromosome 5B (*ZIP4‑5B*). The three Group 3 copies resemble each other closely, as expected of related (homoeologous) genes, but the 5B copy tells a different evolutionary story. Sequence comparisons revealed that *ZIP4‑5B* originated from a duplication of the 3B copy, most likely during wheat polyploidization, followed by divergence, particularly in the N‑terminal region [51]. This diverged copy was sitting precisely within the classical *Ph1* interval.

Expression data sharpened the picture. Whereas the Group 3 *ZIP4* copies show the modest, meiosis‑specific expression patterns characteristic of meiotic genes, *ZIP4‑5B* is expressed strongly during meiosis and more broadly across tissues [12,27]. In meiocytes, *ZIP4‑5B* expression is clearly higher than that of the Group 3 *ZIP4* copies, and there is no compensatory increase in expression of these Group 3 *ZIP4* copies in the *ph1b* mutant, in which *ZIP4‑5B* has been deleted [12,27]. Together, these observations suggested that the *ZIP4‑5B* copy had acquired a distinct regulatory context, one compatible with a new and possibly dominant meiotic role.

### (b) Demonstrating that *ZIP4‑5B* underlies the *Ph1* effect

Demonstrating that *ZIP4‑5B* is responsible for the *Ph1* phenotype required functional evidence. Here, the availability of TILLING mutants, high‑quality genome assemblies and CRISPR gene editing was transformative. Mutations in *ZIP4‑5B* (*zip4-5B* mutants), whether chemically induced in TILLING populations or engineered using CRISPR, consistently reproduced the defining meiotic features of *ph1b* mutants [12,45,52]. In hybrids between wheat and wild relatives, loss of *ZIP4-5B* resulted in a sevenfold increase in CO between related chromosomes, compared with when *ZIP4-5B* was present [12,45,52]. This level of CO is indistinguishable from that reported in hybrids between the Sears *ph1b* 59.3 Mb deletion mutant and wild relatives, confirming that *ZIP4-5B* is responsible for the ability of *Ph1* to suppress CO between related chromosomes [12,45,52]. In wheat itself, *zip4-5B* mutants displayed univalents and multivalents at metaphase I, reflecting disrupted homologous recognition and impaired synapsis, again mirroring the *ph1b* phenotype but without the confounding effects of the 59 Mb genomic deletion.

### (c) Two meiotic functions combined in a single gene

Meiotic COs in plants are generated via two main pathways, known as *class I* and *class II* CO pathways [9]. Class I COs account for the majority of COs (approximately 85%) and are subject to interference, limiting the proximity of adjacent COs along a chromosome. By contrast, class II COs occur at lower frequency and are not sensitive to interference. Class I COs rely on a conserved set of proteins known as the ZIP (zipper proteins), Mer (meiotic recombination proteins) and Msh (MutS homologues - DNA mismatch repair proteins) (ZMM) group, which stabilize early recombination intermediates and promote their maturation. The ZIP4 protein is one of these ZMM factors [15].

In budding yeast, the ZIP4 protein links recombination to synaptonemal complex formation by connecting recombination proteins with the chromosome axis; consequently, loss of *ZIP4* completely disrupts synapsis and CO formation [48,53]. In diploid plants, such as *Arabidopsis* and rice, loss of *ZIP4* likewise removes most class I COs (70–85%), although synapsis proceeds largely unaffected [49,50,54]. In contrast to these dramatic effects, cytological analyses in wheat indicate that loss of the *ZIP4-5B* copy results in more specific defects in synapsis and CO formation. This attenuation is likely owing to the presence of the remaining ancestral Group 3 *ZIP4* copies, which partially compensate for the loss of *ZIP4‑5B*.

*Two key meiotic functions of *ZIP4-5B* became apparent*. The first is the promotion of timely synapsis between homologous chromosomes [11,55]. In wild‑type wheat, homologues efficiently recognize and synapse during the stage of the telomere bouquet, when telomeres cluster together at the onset of meiosis. In *zip4‑5B* mutants, synapsis is delayed, so that more synapsis occurs after the telomere bouquet has dispersed, when synapsis can also occur between related chromosomes [11,55]. This delay provides a simple and elegant explanation for why *zip4‑5B* mutants show univalents and multivalents: the critical window for exclusive homologue recognition has been missed.

The second key function for *ZIP4‑5B* is its suppression of COs between related chromosomes, via an action occurring later in recombination. This key insight came from analysing MLH1 (MutL homologue 1 - DNA mismatch repair protein), one of the last proteins to be loaded onto class I recombination intermediates before their maturation into COs [56]. In wheat-rye hybrids, consisting only of related chromosomes, substantial numbers of MLH1‑loaded sites were observed whether or not *Ph1* (*ZIP4‑5B*) was present [56]. This showed that early recombination steps and MLH1 loading were occurring independently of *ZIP4‑5B*. Yet in the presence of *ZIP4‑5B*, virtually none of the MLH1‑marked intermediates resolved into COs. By contrast, when *ZIP4‑5B* was absent, roughly 50% did mature into COs [56]. This demonstrates that *ZIP4‑5B* imposes a late-stage block on the maturation of COs between related chromosome intermediates.

Together, these two functions, namely promotion of early homologue synapsis and prevention of late COs between related chromosomes, create a robust, two‑layered system underpinning the strikingly diploid-like behaviour of wheat during meiosis.

### (d) A separation‑of‑function mutant that preserves fertility

The ability to generate precise mutants affecting *ZIP4‑5B* function finally allowed us to examine the effects of this gene on wheat chromosome behaviour, genome stability and overall wheat fertility. The impact of *ZIP4‑5B* loss on meiosis was profound, despite the presence of the remaining three Group 3 ancestral *ZIP4* copies. Over 50% of meiocytes contained univalents or multivalents, anaphase I frequently showed lagging chromosomes, and around 50% of tetrads carried micronuclei [51]. These micronuclei reflected chromosomes that had failed to segregate and which were subsequently lost, producing smaller pollen grains than normal. Consistent with this, approximately 50% of pollen grains from *zip4‑5B* CRISPR lines were small or immature, and grain number dropped by more than 40%. Female fertility was similarly affected [51].

These consequences extended across generations. Progeny of *zip4‑5B* CRISPR mutants rapidly accumulated chromosome rearrangements and aneuploidies, indicating that loss of *ZIP4‑5B* destabilized the genome itself (figure 1). These findings have recently been independently confirmed in a Chinese study exploiting their own *zip4-5B* mutant. This study reports rapid and extensive genome instability in hexaploid wheat following loss of *ZIP4-5B*, including chromosome rearrangements and the associated chromosome loss and gain [57]. This instability strongly suggests that the duplicated 5B copy arose early during wheat polyploidization, where it likely contributed to the establishment of stable chromosome behaviour in nascent polyploids [51].

Insight into how *ZIP4‑5B* performs its meiotic role also comes from its molecular structure. ZIP4 proteins contain multiple tetratricopeptide repeat (TPR) motifs, forming α‑solenoid scaffolds typical of proteins involved in assembling or organizing large macromolecular complexes [51,53]. Comparative sequence analyses identified an exceptionally conserved residue, Ala167, within a central TPR domain. Strikingly, a TILLING mutant, carrying a single A167V substitution in *ZIP4‑5B,* showed a phenotype very different from full loss‑of‑function alleles [58]. This line, later named *zip4‑ph1d*, permitted COs between related chromosomes when crossed with wild relatives, indicating loss of suppressive function, yet retained nearly normal meiotic stability in wheat itself: only homologous bivalents persisted at metaphase I, micronuclei were rare, pollen size distribution resembled wild type and grain set was unaffected [58]. Thus, the line retains the ability to promote early homologous synapsis during the telomere‑bouquet stage but has lost the ability to block the maturation of COs between late recombination intermediates on related chromosomes [58].

This separation‑of‑function *zip4‑ph1d* phenotype revealed that the two key meiotic roles of *ZIP4‑5B*, namely the promotion of timely homologous synapsis and the suppression of COs between related chromosomes, can be genetically uncoupled. The fact that the *zip4‑ph1d* mutant remains fully fertile, compared with the loss of fertility in full *zip4‑5B* mutants, demonstrates that the promotion of early homologous synapsis is the function most critical for the maintenance of wheat genome stability and fertility. It also suggests an evolutionary trajectory in which the duplicated 5B copy of *ZIP4* initially optimized the timing of synapsis, while only later acquiring the ability to restrict COs between related chromosomes. The variation in CO suppression between related chromosomes observed in some tetraploid hybrids may support this idea [59].

### (e) Applied significance: *zip4‑5B* mutants as tools for pre‑breeding

The identification of *ZIP4‑5B,* and the development of targeted mutants isolating the behaviour of this single gene, have created powerful new tools for wheat pre‑breeding, providing a much cleaner method of modulating recombination between related chromosomes. However, as occurs with *ph1b* [27], complete loss of *ZIP4‑5B* causes substantial meiotic defects, reducing pollen viability and grain number, and leading to rapid accumulation of chromosome rearrangements (figure 1) [51]. This instability therefore limits the use of full loss‑of‑function alleles in elite breeding backgrounds.

This challenge is overcome with the use of the specific *zip4‑ph1d* mutant, in which the ability of *ZIP4‑5B* to suppress COs between related chromosomes is selectively disrupted, while its key role in promoting early homologous synapsis is retained. As a result, the *zip4‑ph1d* mutant enables recombination with wild relatives at levels comparable to other *zip4‑5B* mutants, while remaining fully fertile and cytologically stable in wheat itself, as required for deployment in mainstream breeding programmes. The ability of *zip4‑ph1d* to facilitate introgression while maintaining genomic integrity makes it particularly attractive for pre‑breeding efforts aimed at expanding the narrow genetic base of wheat, incorporating stress‑tolerance traits and accelerating the usage of exotic germplasm (figure 2).

**Figure 2.**
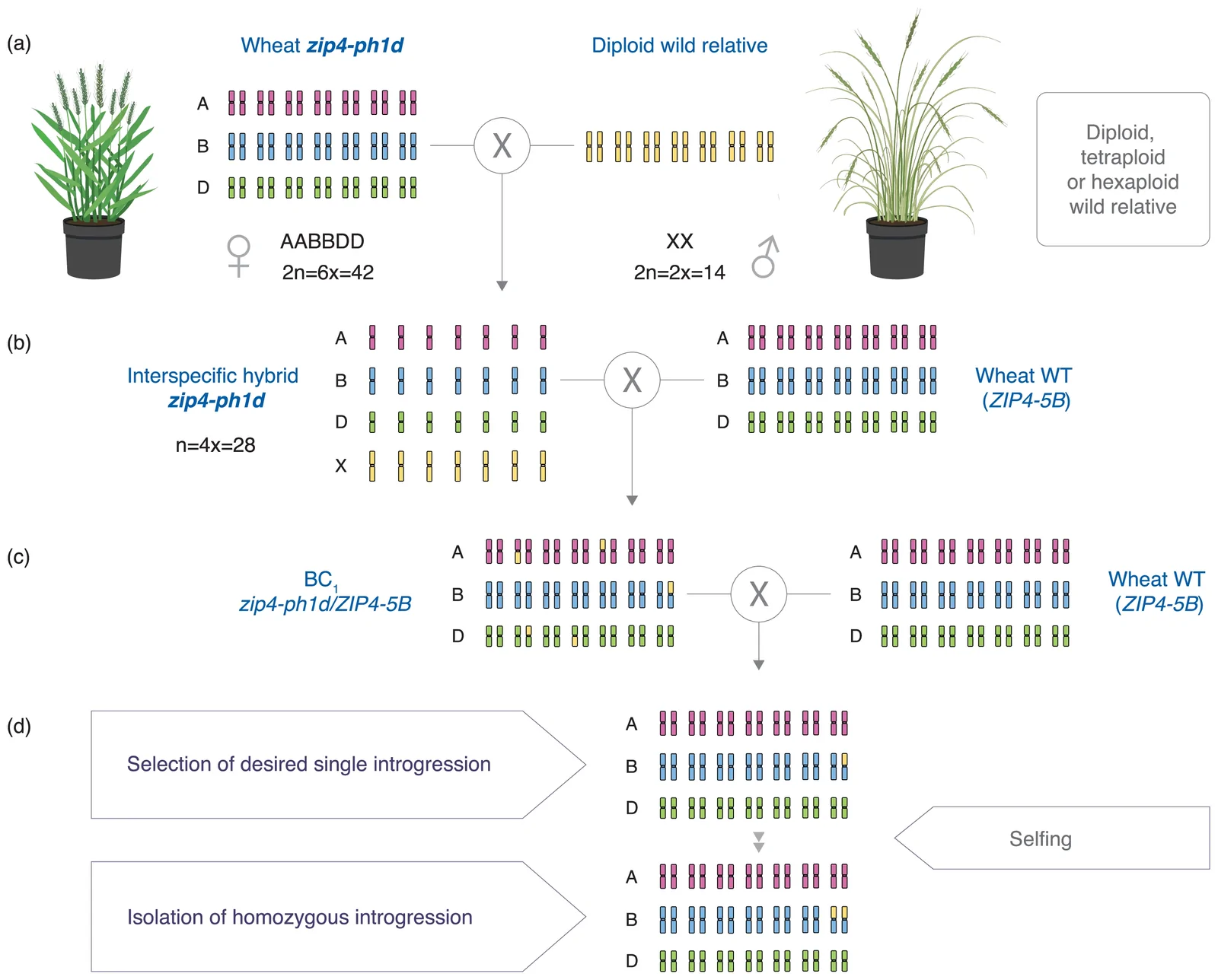
Use of the *zip4-ph1d* mutant to facilitate controlled introgression from wild relatives into wheat. (a) Stable hexaploid wheat carrying the *zip4-ph1d* separation-of-function allele is crossed with a wild relative. In this example, the wild species is diploid, although relatives of other ploidy levels may also be used. (b) In the resulting interspecific hybrid, the *zip4-ph1d* mutation permits COs between wheat chromosomes and those of the wild relative during meiosis. This hybrid is then backcrossed to wild-type wheat (*ZIP4-5B*). (c) Backcrossing to wheat (one or multiple times) restores the wheat chromosome complement while retaining introgressed chromosomal segments derived from the wild relative. (d) Subsequent selection and selfing allow the fixation of specific introgressions as homozygous wheat lines with stable meiosis and full fertility.

## 4. What wheat teaches us about polyploid meiosis

Polyploidy is common across the tree of life from flowering plants to amphibians, fish and budding yeast [10]. It has been especially influential in plants [60], with the majority of species, including major crops, carrying signatures of ancient whole genome duplications [61]. Yet newly formed polyploids face a fundamental challenge: to remain fertile, chromosomes must identify and recombine with their true homologues, despite the presence of multiple closely related partners.

Two major types of polyploids are recognized: autopolyploids and allopolyploids. Autopolyploids arise through the duplication of chromosome sets within a single species. Recent genome-wide association studies have highlighted several candidate meiotic genes that may contribute to meiotic stabilization in autopolyploids [62–64]. Allopolyploids, by contrast, result from the hybridization of different species followed by genome duplication, as is the case for tetraploid and hexaploid wheat. In these species, which combine divergent ancestral genomes, the risk of incorrect chromosome interactions during meiosis is particularly high. The discussion below therefore focuses primarily on allopolyploids.

### (a) Early organization of chromosomes and the limits of sequence‑based homology searching

A central puzzle in polyploid biology is how chromosomes recognize their true partners when many look-alikes coexist in the same nucleus. Classical models of homologue recognition derive largely from budding yeast and *Arabidopsis*, where small genome sizes have favoured models centred on sequence‑dependent interactions along chromosome axes [65]. However, if homologue recognition relied solely on extensive sequence searching, the time required for homologue recognition would be expected to increase with genome size. Yet strikingly, meiosis in wheat, despite its genome being approximately 1000‑fold larger than that of budding yeast, does not progress dramatically more slowly, suggesting the involvement of additional mechanisms [66,67]. Indeed, studies in wheat revealed that early chromosome organization provides crucial structural cues that promote efficient and accurate homologue recognition.

In most plant cells, including wheat, chromosomes adopt a Rabl configuration, being doubled back on themselves with telomeres organized in one half of the nucleus and centromeres in the other. At the onset of meiosis, telomeres cluster more closely together, forming a single telomere ‘bouquet’ [68], helping homologues to find their partners, and initiating associations and synapsis.

It was initially believed that telomere association represented the first step in meiosis. However, studies in hexaploid wheat showed that during pre-meiosis, just after chromosome replication, centromeres associate into pairs, forming several large clusters at the start of meiosis when the telomeres are also clustering [69]. Although most of these early associations are non-homologous, the proportion that are homologous is significantly higher than expected by chance. This behaviour is far more pronounced in hexaploid wheat than in its diploid relatives, suggesting that polyploidization enhanced early centromere dynamics as a preliminary homologue‑sorting mechanism [70]. Subsequent studies in budding yeast and then other plant and animal species showed that, at the onset of meiosis, centromeres associate as pairs independently of telomeres [71].

During the telomere bouquet, telomeres bring chromosome ends together, creating a favourable geometry for homologous interactions. Wheat chromosomes then undergo synchronized chromatin decondensation, beginning at telomeric regions and progressing toward the centromeres. Homologues (>99% identity) remodel their chromatin in near-perfect synchrony, whereas more diverged related chromosomes show progressively less synchronized behaviour [72,73]. This synchrony acts as a rapid, genome‑scale recognition cue and has now been observed in diploid systems, such as *Caenorhabditis elegans* [74], indicating that wheat has adapted an ancient homology‑sensing mechanism to the demands of polyploidy.

In wheat, only homologues can synapse during the telomere bouquet stage, whereas related chromosomes cannot [11]. However, after telomere bouquet dispersal, synapsis becomes less restrictive, allowing related chromosomes to synapse if homologue synapsis is incomplete. Thus, the timing of homologous synapsis during the telomere bouquet is critical to avoid later mispairing. Unlike in diploids, disruption of early homologue recognition in polyploid wheat reveals a later, less stringent process available for synapsis between related chromosomes [11].

Wheat therefore reveals that polyploid stability depends not only on homology *per se* but also on the temporal choreography of synapsis.

### (b) *ZIP4‑5B* as a regulator linking synapsis and recombination

Elegant cytological studies in the fungus Sordaria showed that the ZIP4 protein loads along the whole chromosome axis during early meiosis [75]. ZIP4 sites hanging off these axes link with corresponding sites on partner chromosomes to form cross-bridges, allowing the homologues to align. Thus, during meiosis, ZIP4 sites occur in regions from which homologue alignment is initiated and from which chromosome synapsis begins. In Sordaria and budding yeast, loss of *ZIP4* reduces chromosome synapsis and results in CO failure [48,75].

In hexaploid wheat, carrying four *ZIP4* genes (three Group 3 genes and the additional *ZIP4‑5B* copy), deletion of the single *ZIP4-5B* copy was enough to delay homologous synapsis [11], consistent with its role in accelerating chromosome alignment during the telomere bouquet stage, when synchronized chromatin dynamics still provide a strong recognition cue. In tetraploid wheat, which carries only three *ZIP4* genes (two Group 3 and the *ZIP4‑5B* copy), analysis of multiple mutant combinations demonstrated that deletion of all three *ZIP4* copies delayed synapsis, whereas deletion of only the two ancestral Group 3 copies had no major effect [55]. It is possible that these wheat ancestral Group 3 copies may be behaving similarly to that in *Arabidopsis* and rice, in which loss of *ZIP4* appears to have little impact on chromosome synapsis [49,50].

In terms of CO formation, disruption of the two Group 3 *ZIP4* copies in tetraploid wheat causes approximately a 76–78% reduction in COs, similar to the reduction in class I COs observed in other species when *ZIP4* has been disrupted [55]. This supports the suggestion that these tetraploid wheat Group 3 copies behave more like those found in *Arabidopsis* and rice. Conversely, when all three tetraploid wheat *ZIP4* copies (including *ZIP4-5B*) were eliminated, COs were reduced by more than 95%, indicating that *ZIP4-5B* may affect not only class I COs, but also class II CO pathways [55]. This implies that, in wheat, the class I and class II pathways, usually considered independent [54], may be functionally linked. It also suggests that duplication of *ZIP4* enabled control over the maturation of nearly all recombination intermediates into COs, an important prerequisite for polyploid stability and fertility. Thus, although *ZIP4‑5B* does not prevent synapsis between related chromosomes after telomere bouquet dispersal, when the stringency and correction of chromosome alignment are reduced, it does prevent MLH1‑marked recombination intermediates involving related chromosomes from maturing into COs [56]. As a consequence, related chromosomes that have undergone synapsis ultimately segregate without CO connections. The observation that approximately 50% of wheat metaphase I cells contain bivalents with only a single CO, rather than one on each arm, suggests that a low level of incorrect synapsis still occurs despite the ability of *ZIP4-5B* to promote correct chromosome alignment. However, the uncoupling of MLH1 loading from COs maturation reveals that, unlike in diploids, a second distinct quality‑control step operates in large polyploid genomes such as wheat.

Collectively, these results show that the *ZIP4-5B* copy acquired an additional meiotic function during wheat polyploidization, reinforcing both synapsis and CO control. Together, these activities establish *ZIP4-5B* not simply as one meiotic factor among many, but as a regulatory hub coordinating the timing of homologous synapsis with the selective resolution of recombination intermediates. This dual role appears to represent a key evolutionary innovation enabling meiotic stability in a large and complex polyploid genome.

### (c) Broader insights from wheat

As described above, the wheat system has generated conceptual advances with applications extending beyond cereals into the general biology of meiosis, including:

— the identification of pre-meiotic centromere pairing;
— the importance of early homologue chromatin synchronization in large genomes;
— the clarification that timing of synapsis is a fidelity mechanism, not merely a structural process;
— the finding that MLH1 loading does not always predict CO formation, challenging assumptions derived from diploid models; and
— the discovery that duplicated meiotic genes can evolve new functions, enabling rapid stabilization of a newly formed polyploid genome.

These findings show that large polyploid genomes rely on structural and temporal cues, not solely DNA sequence homology, to achieve accurate pairing. They require refined regulatory strategies to maintain meiotic fidelity. Wheat demonstrates that polyploid meiosis is built not on new components, but on subtle evolutionary modifications to conserved mechanisms, precisely tuned to the demands of genome size and chromosome complexity.

## 5. Concluding remarks and future challenges

The work reviewed here shows that the extraordinary stability and fertility of polyploid wheat is supported by precisely tuned meiotic controls rather than by a fundamental redesign of the meiotic programme. Identification of *ZIP4‑5B* as the molecular basis of the long‑studied *Ph1* locus has provided a mechanistic explanation for how wheat achieves diploid‑like meiosis despite carrying multiple related chromosome sets. By coordinating the timing of homologous synapsis with late‑stage CO control, this duplicated meiotic gene ensures faithful recombination, preserves genome integrity and ultimately sustains fertility.

Hexaploid wheat is not a model system, given its long life cycle, multiple genomes possessing multiple related chromosomes and total genome size 1000-fold larger than that of budding yeast. Yet, as described in the previous section, studies in wheat have made important contributions to the general field of meiosis.

### (a) Unresolved mechanistic questions

Despite these advances, key mechanistic questions remain unanswered. The molecular basis by which *ZIP4‑5B* orchestrates synapsis timing and CO control remains unknown. In particular, the protein–protein interactions mediated by the ZIP4‑5B protein scaffold, how these differ from those of the ancestral Group 3 *ZIP4* copies and how ZIP4‑5B interacts with other components of the recombination machinery—such as the mismatch repair protein MSH7 (formerly termed *Ph2*) [76]—remain to be elucidated. The observation that elimination of all *ZIP4* copies in tetraploid wheat suppresses more than 95% of COs suggests that class I and class II CO pathways, traditionally treated as independent, may be functionally linked in wheat. Defining how this coordination is achieved and whether it represents a general feature of large polyploid genomes, is an important challenge for future studies.

Wheat has also revealed that homologue recognition and synapsis can be temporally separated. Disruption of early homologue recognition during the telomere‑bouquet stage exposes a later, less stringent synapsis process that can take place between related chromosomes. Whether this represents a conserved backup system that preserves some level of chromosome segregation when early synapsis fails, or a polyploid‑specific adaptation that emerged during genome duplication, remains an open question.

A closely related challenge is understanding the mechanistic basis of homologue recognition during the telomere‑bouquet stage in large, complex genomes. In wheat, early meiosis is marked by synchronized chromatin decondensation that initiates at telomeres and progresses towards centromeres, with true homologues (>99% identity) showing near‑perfect synchrony. This suggests that chromatin dynamics provide a key homology cue early in meiosis. However, it is unclear how homologues coordinate these changes, whether this process is driven by sequence identity, shared epigenetic and transcriptional states, nuclear positioning or a combination of these factors and how it is integrated with telomere‑mediated chromosome movements. Elucidating the mechanism underlying synchronized chromatin remodelling will be critical for understanding how homologue recognition is achieved rapidly and reliably in large polyploid genomes.

### (b) Translating meiotic insight into breeding strategies

The discovery of *ZIP4‑5B* has also generated practical opportunities for wheat improvement. For over four decades, the classical *ph1b* deletion mutant has been widely used to promote recombination between wheat and its wild relatives, enabling the transfer of valuable agronomic traits (reviewed in [77]). However, the large chromosomal deletion associated with *ph1b* often leads to reduced fertility, genome instability and the accumulation of undesirable rearrangements (figure 1). The *zip4‑ph1d* separation‑of‑function mutant overcomes many of these limitations by selectively permitting COs between related chromosomes while preserving early homologous synapsis, genome stability and full fertility (figure 2). As such, *zip4‑ph1d* provides a substantially improved tool for pre‑breeding programmes, allowing more efficient introgression with reduced risk of background genomic disruption.

Complementary approaches may further enhance the controlled use of homoeologous recombination. Not all wheat–alien chromosome combinations are regulated equally by *Ph1*. Several alien chromosomes have long been known to suppress *Ph1* activity and promote recombination between wheat and related chromosomes, including chromosome 5U from *Aegilops umbellulata,* chromosome 5E from *Elytrigia elongate* and chromosome 3S from *Aegilops speltoides* [78–80]. More recently, chromosome 5 Mg from *Aegilops geniculata* was shown to promote high levels of recombination with wheat chromosomes even in the presence of *Ph1* [81]. Exploitation of such systems, particularly in combination with *ph1* or *zip4-5B* mutants, can increase recombination across broader chromosome regions than is typically observed in wheat and provides a valuable complementary strategy for introgression breeding [82]. Magnesium supplementation has also been shown to increase CO formation between related chromosomes in appropriate genetic backgrounds [45].

Although these approaches can increase the frequency of homoeologous recombination, a major remaining challenge is their limited ability to alter where COs occur along the chromosome. In both homologous and related chromosome pairs, COs remain strongly concentrated in distal regions, leaving large portions of the chromosome largely inaccessible to recombination [83–87]. Overcoming this constraint and achieving more even or targeted CO distributions would substantially increase the efficiency of introgression and genomic reshaping during pre‑breeding.

Maintaining meiotic fidelity under changing environmental conditions represents an equally important applied challenge. Wheat meiosis is particularly sensitive to temperature with short periods of heat stress during early prophase I disrupting recombination and reducing grain number [88]. Recent work indicates that components of the recombination machinery, including *DMC1*, contribute to stabilizing CO formation across temperature extremes [89]. Understanding how *ZIP4‑5B* and related pathways respond to environmental stress and whether their activity can be buffered or modulated will be critical for sustaining reproductive success under future climate conditions.

Together, the work discussed here highlights how the wheat polyploid genome achieved stable meiosis, not by reinventing the meiotic programme, but by refining its temporal and regulatory control. In this context, meiotic control in polyploid wheat does not simply preserve genome stability but also defines how much genetic diversity can be introduced and maintained without compromising fertility. At a time when wheat improvement increasingly integrates artificial intelligence and machine‑learning approaches with large‑scale genomic and phenotypic datasets, the limiting factor is no longer the identification of useful variation, but its successful incorporation into elite germplasm. Wild relatives and historic landraces harbour traits essential for future adaptation, including disease resistance and tolerance to heat and drought under changing climates [90]. Understanding how meiotic processes regulate genetic exchange therefore provides the biological foundation needed to fully exploit these emerging technologies.

## Data Availability

This article has no additional data.
